# Association of cognitive reserve with transitions across cognitive states and death in older adults: A 15‐year follow‐up study

**DOI:** 10.1002/alz.13910

**Published:** 2024-05-23

**Authors:** Yuanjing Li, Serhiy Dekhtyar, Giulia Grande, Grégoria Kalpouzos, Caterina Gregorio, Erika J. Laukka, Chengxuan Qiu

**Affiliations:** ^1^ Aging Research Center, Department of Neurobiology, Care Sciences and Society Karolinska Institutet‐Stockholm University Solna Sweden; ^2^ Biostatistics Unit, Department of Medical Sciences University of Trieste Trieste TS Italy; ^3^ MOX ‐ Modeling and Scientific Computing Laboratory, Department of Mathematics Politecnico di Milano, Piazza Leonardo da Vinci Milano Italy; ^4^ Stockholm Gerontology Research Center Stockholm Sweden

**Keywords:** Cohort study, Dementia, Lifelong cognitive reserve, Mild cognitive impairment, Pathological brain aging

## Abstract

**INTRODUCTION:**

We investigated the association of cognitive reserve (CR) with transitions across cognitive states and death.

**METHODS:**

This population‐based cohort study included 2631 participants (age ≥60 years) who were dementia‐free at baseline and regularly examined up to 15 years. Data were analyzed using the Markov multistate models.

**RESULTS:**

Each 1‐point increase in the composite CR score (range: ‐4.25 to 3.46) was significantly associated with lower risks of transition from normal cognition to cognitive impairment, no dementia (CIND) (multivariable‐adjusted hazards ratio = 0.78; 95% confidence interval = 0.72–0.85) and death (0.85; 0.79–0.93), and from CIND to death (0.82; 0.73–0.91), but not from CIND to normal cognition or dementia. A greater composite CR score was associated with a lower risk of transition from CIND to death in people aged 60‐72 but not in those aged ≥ 78 years.

**DISCUSSION:**

CR contributes to cognitive health by delaying cognitive deterioration in the prodromal phase of dementia.

**Highlights:**

- We use Markov multistate model to examine the association between cognitive reserve and transitions across cognitive states and death.
- A great cognitive reserve contributes to cognitive health by delaying cognitive deterioration in the prodromal phase of dementia.
- A great cognitive reserve is associated with a lower risk of transition from cognitive impairment, no dementia to death in people at the early stage of old age, but not in those at the late stage of old age.

## 1 INTRODUCTION

Stimulating experiences over the lifespan, such as early‐life educational attainment, adulthood socioeconomic position and occupational complexity, and late‐life social engagement, may all contribute to the cognitive reserve (CR).^1^ In recent years, evidence from population‐based studies has accumulated that higher CR is associated with a reduced risk of cognitive decline, mild cognitive impairment (MCI), and dementia in late life.^2^, ^3^, ^4^, ^5^ When studying the transitions across cognitive states from normal cognition through prodromal phase of dementia (e.g., MCI) to clinical dementia, a critical issue that is often missed is the fact that transitions are not unidirectional in the prodromal state of dementia.^6^, ^7^, ^8^ This is important because prospective cohort studies have suggested that a considerable proportion of individuals experience fluctuations across cognitive states in late life, such as regression from MCI to normal cognitive function and progression from normal cognition or MCI to dementia.^6^, ^7^, ^8^ The dynamic bidirectional transitions of cognitive states shall be taken into account when assessing late‐life changes in cognitive health. In addition, it is important to consider the transition from different cognitive states to death because cognitive impairment is also associated with increased mortality in older people.^9^

Previous studies have suggested that the potential associations of individual CR proxies with dementia and MCI might vary by demographic features and genetic susceptibility (i.e., *apolipoprotein E [APOE]* genotypes).^10^, ^11^, ^12^ For instance, data from the Mayo Clinic Study of Aging have indicated that a higher CR proxy (i.e., late‐life social activity) is associated with a lower risk of MCI in *APOE*‐ε4 carriers, but not in noncarriers.^12^ However, the interplay of CR with demographic factors and *APOE* genotypes on the transitions across various cognitive states and death remains unclear. Finally, conceptually, higher CR is associated with better cognitive function at a given load of underlying neuropathology.^13^ Therefore, brain pathology should be taken into account when operationalizing CR and examining its association with cognitive phenotypes. A previous study addressed this issue by adjusting for whole‐brain gray matter (GM) volume relative to total intracranial volume in order to capture brain atrophy.^14^ In addition, microvascular damage is highly relevant for cognitive decline and dementia and, therefore, should be considered in assessing the role of CR in cognitive function.^15^, ^16^

**RESEARCH IN CONTEXT**

1. **Systematic review**: We searched PubMed for literature. Emerging evidence has linked cognitive reserve (CR) with cognitive impairment, no dementia (CIND) and dementia. However, the associations of CR with transitions across cognitive states (e.g., from CIND to normal cognition) and death are still unclear. Furthermore, the roles of age, sex, and *apolipoprotein E (APOE)* genotypes in such associations have yet to be explored.
2. **Interpretation**: This 15‐year cohort study of Swedish older adults showed that a great CR was associated with reduced likelihood of transition from normal cognition to CIND, and from CIND to death, but not from CIND to normal cognition or dementia, or from dementia to death. When controlling for structural brain aging biomarkers, the reduced risk of transitions from normal cognition to CIND or death associated with great CR remained, while the risk of transition from CIND to death became nonsignificant. Furthermore, the association of great CR with reduced risk of transition from CIND to death was evident in people aged 60‐72 years but not in those aged ≥78 years.
3. **Future directions**: Future large‐scale prospective cohort studies should investigate the influence of CR on cognitive transitions among ethnically and socioculturally diverse populations as well as neuropathological mechanisms underlying the transitions.

Therefore, in this population‐based cohort study of Swedish older adults, we sought to examine the associations between CR and transitions across cognitive states and death, while taking into account potential variations by age, sex, and *APOE* genotypes. We further assessed the associations by taking into account MRI biomarkers of cerebral atrophy and microvascular damage. We hypothesized that individuals with higher composite CR may have a lower risk of transition from normal cognitive function through prodromal dementia to clinical dementia or death, and a higher risk of transition from prodromal dementia to normal cognitive function and that the transitions would be independent of neuropathological markers and more prominent among the *APOE*‐ɛ4 allele carriers than noncarriers.

## 2 METHODS

### 2.1 Study design and participants

**FIGURE 1:**
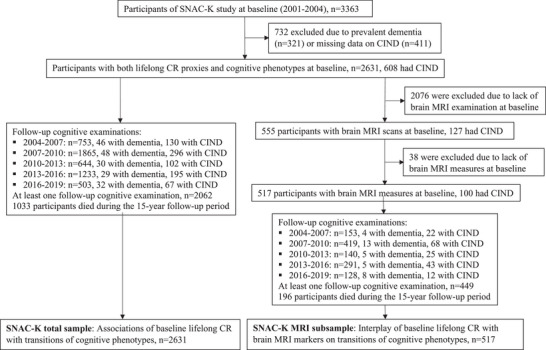
Flowchart of study participants. CIND, cognitive impairment, no dementia; CR, cognitive reserve; MRI, magnetic resonance imaging; SNAC‐K, The Swedish National study on Aging and Care in Kungsholmen.

This population‐based cohort study used data from the Swedish National study on Aging and Care in Kungsholmen (SNAC‐K) and the magnetic resonance imaging (MRI) substudy, as previously reported.^17^ In brief, SNAC‐K is an ongoing population‐based longitudinal study of aging and health among people aged 60 years and older in the Kungsholmen district of central Stockholm, Sweden. In 2001‐2004, 3363 individuals underwent baseline examinations. The follow‐up examinations were performed every 6 years for people aged 60, 66, and 72 years and every 3 years for those aged 78 years and above, until 2016‐2019. Out of the 3363 participants at baseline, 732 were excluded due to the diagnosis of prevalent dementia (*n* = 321) or insufficient information from the cognitive battery for defining CIND (*n* = 411), leaving 2631 participants for analysis regarding the association of composite CR with cognitive transition (the total sample). A subsample consisting of 555 noninstitutionalized and nondisabled participants underwent structural brain MRI examinations in 2001‐2003; Of these, 38 were excluded due to incomplete or suboptimal quality of images, brain infarcts, brain tumors, or arachnoid cysts, leaving 517 persons for assessing the association of composite CR with cognitive transmissions while considering MRI markers of structural brain aging (the MRI subsample). Figure 1 shows the flowchart of study participants.

All phases of data collection in SNAC‐K were approved by the Ethics Committee at Karolinska Institutet or the Regional Ethical Review Board in Stockholm.

### 2.2 Data collection and assessments at baseline

At baseline, we collected data on demographics (age, sex, and education), lifestyle (e.g., smoking and alcohol consumption), cardiometabolic conditions (e.g., body mass index, blood pressure, blood cholesterol, and glycated hemoglobin), chronic diseases (e.g., heart failure), and cognitive function through in‐person interviews, clinical examinations, laboratory test, and cognitive testing, as previously described.^18^

#### 2.2.1 Assessment of CR

The assessments of lifelong factors associated with CR have been previously reported.^19^ In brief, data on early‐life education were collected by inquiring about the total years of formal schooling. Data on midlife work complexity were collected by inquiring about the five longest‐held occupations of participants, which were then ranked based on their substantive complexity using a continuous score ranging from 0.7 to 10. A higher score indicates greater substantive complexity.

Data on late‐life leisure activities were assessed by asking participants if they had engaged in any of 26 listed activities and the frequency of their participation in the past year. These activities were grouped into three components: mental (i.e., reading newspapers or books, watching TV, playing chess, cards, musical instruments, or computer games, or listening to music), social (i.e., sport engagement, dance, bingo, traveling, visiting cinema, theater, concert, museum, art exhibition, restaurant, or church, taking course, or participating in nonprofit activities or association work), and physical components (i.e., gardening, forest roaming, hunting, fishing, knitting, weaving or sewing, painting or drawing, or repairing house, car, or other machine instrument). The extent of engagement in the mental and social components was determined by the number of activities participated in and categorized as low (≤1 activity), moderate (2‐3 activities), or high (≥4 activities), with corresponding scores of 0, 1, and 2. The extent of engagement in the physical component was assessed by the frequency of participation and categorized as low (less than once per week), moderate (once per week), or high (more than once per week), with corresponding scores of 0, 1, and 2. The leisure activity score was calculated by summing the scores of the three components, with a range from 0 to 6, where a higher score indicates greater engagement in leisure activities.^19^

The late‐life social network was assessed in terms of size and support. The size of late‐life social networks was assessed through several indicators, including marital status, living arrangement, number of children, frequency of contact with relatives or friends, and the number of contacts.^19^ In addition, measures of perceived material and psychosocial support received, satisfaction with social connections, and sense of belonging to social groups were also used to assess support of late‐life social networks. All indicators were standardized into z‐scores and separately averaged to estimate network size and support. The late‐life social network score was calculated by averaging the scores of size and support, ranging from −2.1 to 1.2, with a higher score indicating a richer social network.^19^

We employed structural equation modeling, guided by a hypothesis‐driven approach, to generate the composite CR score from the four aforementioned indicators that could contribute to CR. With this model, we assessed the causal relationships between the four observed indicators and the underlying latent variable, represented by the composite CR score. Notably, the model exhibited a strong fit (χ2 goodness of fit = 0.88, comparative fit index = 1.00, root mean square error of approximation = 0.001). Finally, we computed the latent composite CR score by summing the products of the four standardized indicators and their respective weights (Supplemental Figure 1).^19^ The composite CR score followed a normal distribution, ranging from −4.25 to 3.46.

#### 2.2.2 Diagnosis of dementia and definition of CIND at baseline and follow‐ups

We used a neuropsychological test battery to assess the function of five cognitive domains,^20^ that is, episodic memory (free recall test), executive function (trail‐making test, part B), language (test of category and letter fluency), visuospatial abilities (test of mental rotations), and perceptual speed (digit cancellation test and pattern comparison test). Global cognitive function was assessed using the Mini‐Mental State Examination (MMSE) test.

Dementia was clinically diagnosed according to the Diagnostic and Statistical Manual of Mental Disorders, fourth edition, criteria, following a three‐step procedure.^18^ In brief, an examining physician, who was engaged in data collection, initially formulated a preliminary diagnosis, subsequently complemented by a second preliminary diagnosis conducted by a reviewing physician. The cognitive assessment used for dementia diagnosis included the MMSE test, the clock drawing test, the digit span forward and backward test, and items covering memory, executive functioning, problem‐solving, orientation, and interpretation of proverbs. For any disagreement in the diagnosis between the two physicians, a neurologist external to the data collection was consulted and a final diagnosis was made.^18^ For deceased participants who had not been diagnosed with dementia via follow‐up examinations, the dementia status was ascertained based on medical charts reviewed by physicians in the SNAC‐K research group and through linkage to the Swedish Cause of Death Register.^18^

To define the status of CIND, we first standardized the raw score for each cognitive test into a Z score using the baseline mean and standard deviation (SD). We then calculated the mean Z score for a specific cognitive domain when that domain included more than one test. Among dementia‐free participants, CIND was defined as scoring at least 1.5 SDs below the age group‐specific mean in any of the five examined cognitive domains.^21^

#### 2.2.3 Acquisition and evaluation of MRI data

For all eligible participants, structural brain MRI scans were performed on a 1.5T scanner (Philips Intera, The Netherlands) at baseline. The core MRI sequences and parameters were reported in our previous work.^22^ The MRI measures of brain pathology were meant to capture cerebral atrophy and microvascular damage. We used GM volume, hippocampal volume, and white matter (WM) volume to capture brain atrophy. The GM volume, WM volume, and total intracranial volume were automatically assessed in Statistical Parametric Mapping, using the T1‐weighted images.^22^ The hippocampal volume was automatically assessed in the FreeSurfer 5.1 image analysis suit, using the T1‐weighted images.^3^ Volumes of GM, WM, and hippocampus were adjusted by total intracranial volume using linear regression.^3^ To capture microvascular damage, we used MRI markers of WM hyperintensities (WMH), lacunes, and perivascular spaces. A senior neuroimaging analyst (G.K.) manually drew WMH on fluid‐attenuated inversion recovery (FLAIR) images and interpolated them on corresponding T1‐weighted images to compensate for the gap between slices in a FLAIR sequence.^22^ Then, the global volume of segmented WMH was automatically estimated in the MRIcron. We log‐transformed the WMH volume at baseline to reduce skewness. We did not adjust WMH volume by total intracranial volume because the log‐transformed WMH volume was not significantly associated with total intracranial volume. A trained rater (Y.L.) visually counted the total number of perivascular spaces following a validated protocol,^22^, ^23^ and counted lacunes according to the criteria of the STandards for ReportIng Vascular changes on nEuroimaging (STRIVE),^24^ without knowledge of the participants’ clinical characteristics. We dichotomized lacunes as absence versus presence because the distribution of lacunar count was heavily right‐skewed.

### 2.3 Statistical analysis

We compared the characteristics of study participants by CIND status at baseline using t‐test for continuous variables and *χ* ^2^ test for categorical variables in both the total sample and the MRI subsample. We used scatter plots to show the correlations between composite CR score and structural brain measures. Then, in the SNAC‐K total sample (*n* = 2631), we investigated the association between composite CR score and transitions across cognitive states and death, using the Markov multistate survival model, where four distinct states (normal cognition, CIND, dementia, and death) and initial values for the transition matrix were specified. All transitions across cognitive states and death between two consecutive follow‐up examinations were taken into account. We also examined the association between the four lifespan individual indicators and transitions across cognitive states and death, using the Markov multistate survival model. The follow‐up time (years) was used as the time scale. Furthermore, we used Markov multistate survival model to examine the statistical interactions of age groups (60‐72 years vs. ≥78 years), sex, and *APOE* genotypes (*APOE*‐ε4 allele no vs. yes) with the baseline composite CR score on transitions across cognitive states or death. When a statistical interaction was detected, we further assessed the direction and strength of the association between the composite CR score and transitions across cognitive states or death, stratified by age groups, sex, or *APOE* genotypes. We controlled for age (years), current smoking, heavy alcohol drinking, hypertension, high total cholesterol, diabetes, body mass index, atrial fibrillation, ischemic heart disease, and heart failure, and if applicable, for sex and *APOE* genotypes, in the aforementioned association analyses.

Finally, we repeated these analyses in the MRI subsample (*n* = 517), while additionally adjusting for the MRI markers of cerebral atrophy and microvascular damage at baseline. Stata Statistical Software: Release 16.0 for Windows (StataCorp LLC., College Station, TX) was used for all analyses.

## 3 RESULTS

### 3.1 Baseline characteristics of study participants

At baseline, the mean age of the 2631 participants was 72.3 years (SD: 9.9; range: 60‐101), 61.4% were female, and 23.1% had CIND. The composite lifelong CR score ranged from −4.25 to 3.46 (median, 0; interquartile range, 1.83). Compared to people free of CIND at baseline, those with CIND had lower levels of CR proxies and composite CR score, and were older, more likely to be female, to smoke, and to have hypertension, diabetes, ischemic heart disease, and heart failure, and to carry the *APOE*‐ε4 allele, while less likely to have a heavy alcohol consumption (*P *< 0.02, Table 1). The mean body mass index and distribution of atrial fibrillation did not differ significantly between people with or without CIND at baseline (*P *> 0.05, Table 1). In addition, a higher CR score was significantly correlated with greater volumes of GM and hippocampus and lower WMH volume (*P *< 0.05), but not with the number of perivascular spaces (Supplemental Figure 2).

**TABLE 1:** Characteristics of study participants by cognitive impairment, no dementia at baseline in the SNAC‐K total sample (*n* = 2631).

Characteristics	Total sample, *n* = 2631	Cognitive impairment, no dementia		
		No, *n* = 2023	Yes, *n* = 608	*P‐*Value
Age, years	72.34 (9.94)	71.75 (9.88)	74.29 (9.90)	<0.001
Female, *n* (%)	1614 (61.35)	1212 (59.91)	402 (66.12)	0.006
Education^a^, years	12.32 (4.00)	12.78 (3.95)	10.77 (3.76)	<0.001
Midlife work complexity score^a^	5.00 (1.81)	5.16 (1.80)	4.49 (1.76)	<0.001
Late‐life leisure activity score^a^	2.47 (1.45)	2.58 (1.45)	2.06 (1.38)	<0.001
Late‐life social network score^a^	0.86 (0.51)	0.14 (0.48)	‐0.09 (0.57)	<0.001
Composite cognitive reserve score	0 (1.26)	0.17 (1.22)	‐0.57 (1.24)	<0.001
Current smoking, *n* (%)	368 (13.99)	264 (13.05)	104 (17.11)	0.011
Heavy alcohol drinking, *n* (%)	445 (16.91)	365 (18.04)	80 (13.16)	0.005
Hypertension, *n* (%)	1963 (74.61)	1481 (73.21)	482 (79.28)	0.003
Diabetes, *n* (%)	240 (9.12)	154 (7.61)	86 (14.14)	<0.001
High total cholesterol^a^, *n* (%)				0.003
No	1246 (47.36)	955 (47.21)	291 (47.86)	
Yes	1307 (49.68)	1020 (50.42)	287 (47.20)	
Body mass index, kg/m^2^	25.78 (4.02)	25.84 (3.96)	25.59 (4.23)	0.179
*APOE* genotypes^a^, *n* (%)				<0.001
No ε4 allele	1744 (66.29)	1372 (67.82)	372 (61.18)	
Any ε4 allele	722 (27.44)	549 (27.14)	173 (28.45)	
Atrial fibrillation, *n* (%)	216 (8.21)	156 (7.71)	60 (9.87)	0.089
Heart failure, *n* (%)	201 (7.64)	133 (6.57)	68 (11.18)	<0.001
Ischemic heart disease, *n* (%)	347 (13.19)	244 (12.06)	103 (16.94)	0.002

*Note*: Data were mean (SD), unless otherwise specified.

Abbreviations: *APOE, apolipoprotein E*; SNAC‐K, The Swedish National Study on Aging and Care in Kungsholmen.

^a^ The number of participants with missing value was 18 for education, 19 for midlife work complexity, 264 for late‐life leisure activities, 103 for later‐life social network, 78 in high total cholesterol, and 165 in *APOE* genotype.

In the SNAC‐K MRI subsample, compared to those without CIND, individuals with CIND at baseline (19.3%) had a lower composite CR score, lower educational levels, and less midlife work complexity, and were more likely to have diabetes and heart failure (*P *< 0.05, Supplemental Table 1). There were no significant differences in mean age and body mass index, and in the proportions of females, current smoking, alcohol consumption, hypertension, atrial fibrillation, ischemic heart disease, and *APOE* genotypes between the two groups (*P *> 0.05, Supplemental Table 1).

### 3.2 Association of CR with transitions across cognitive states and death

**FIGURE 2:**
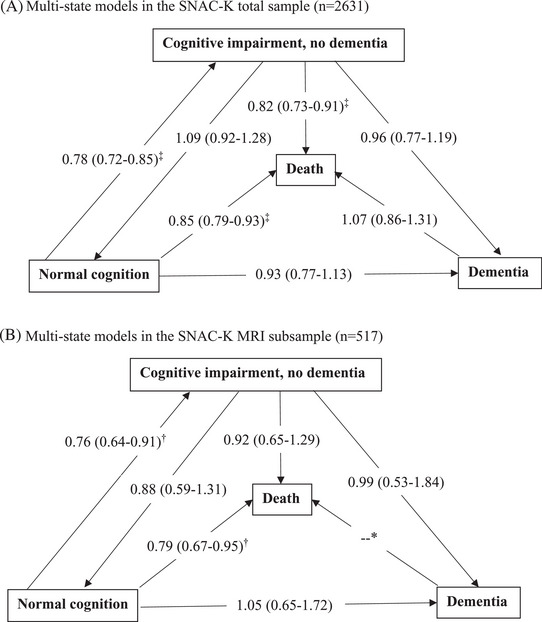
Association of cognitive reserve with transitions across cognitive states and death in the SNAC‐K total sample (A) and the SNAC‐K MRI subsample (B). Data are hazards ratio (95% confidence interval) of the transition across states associated with per 1‐point increase in the composite cognitive reserve score (range: −4.25 to 3.46), derived from the Markov multistate model. MRI, magnetic resonance imaging; SNAC‐K, The Swedish National Study on Aging and Care in Kungsholmen;. ^*^The parameters were not estimated due to the limited sample size. ^†^ *P *< 0.01, ^‡^ *P *< 0.001.

In the SNAC‐K total sample (*n* = 2631), the Markov multistate survival model showed that each 1‐point increase in the composite CR score at baseline was associated with a ∼22% lower risk of transition from normal cognition to CIND and a ∼15% lower risk of transition from normal cognition to death, as well as a ∼18% lower risk of transition from CIND to death, during the up to 15 years of follow‐up period (Figure 2A). Supplemental Figure 3A shows the total numbers of all transitions across cognitive states and death in the SNAC‐K total sample. Notably, compared with people in the total sample, participants who underwent the direct transition from normal cognition to dementia (*n* = 96) were older (mean age: 78.8 vs. 72.3 years, *P* < 0.01) and more likely to be females (66.7% vs. 61.3%, *P* < 0.05). To illustrate the association of composite CR with cognitive transitions, we assumed that two subjects (subject 1 and subject 2) had the same age, sex, health conditions, and cognitive states (normal cognition), but had different composite CR scores. Subjects 1 and 2 had a composite CR score of 3 and 0, respectively. Compared to subject 1, on average the hazard ratio of experiencing the transition from normal cognition to CIND for subject 2 was 2.03.

Similarly, in the SNAC‐K MRI subsample, when accounting for the burden of MRI markers of cerebral atrophy and microvascular damage, each 1‐point increase in the composite CR score was linked to a ∼24% lower risk of transition from normal cognition to CIND and a ∼21% lower risk of transition from normal cognition to death, while no significant association with the transition from CIND to death was observed (Figure 2B). There was no significant association between composite CR score and other transitions across cognitive states and death in the SNAC‐K total sample or the MRI subsample (*P *> 0.05). Supplemental Figure 3B shows the total numbers of all transitions across cognitive states and death in the SNAC‐K MRI subsample.

Of the four individual CR indicators, midlife occupational complexity was associated with a higher likelihood of transition from normal cognition to CIND or death as well as a lower likelihood of transition from CIND to normal cognition (*P *< 0.05, Supplemental Figure 4). In addition, a higher level of early‐life educational attainment was associated with a lower likelihood of transition from CIND to dementia (*P *< 0.05, Supplemental Figure 4). Furthermore, late‐life social networks and leisure activity were both associated with a lower risk of transition from normal cognition or CIND to death (*P *< 0.01, Supplemental Figure 4).

### 3.3 Associations of CR with transitions by *APOE* gene and age groups

In the SNAC‐K total sample, using the Markov multistate survival model, a marginally statistically significant interaction was observed between composite CR score and *APOE* genotypes on the transition from normal cognition to dementia (*P* for interaction = 0.051, Table 2). Stratified analysis indicated that each 1‐point increase in the composite CR score was marginally associated with a ∼30% lower risk of transition to dementia in *APOE*‐ε4 carriers (*P *= 0.089), while no significant association was found in noncarriers (*P *= 0.586). Likewise, a statistical interaction was observed between composite CR score and age groups on the transition from CIND to death (*P* for interaction = 0.010, Table 2), such that a higher composite CR score was significantly associated with a ∼35% lower risk of transition from CIND to death in individuals aged 60‐72 years (*P *< 0.001), while a ∼13% marginally lower risk was observed in those aged 78 years and above (*P *= 0.063). There was no statistical or marginal interaction of composite CR score with *APOE* genotypes or age groups on the risk of other transitions across cognitive states and death (*P* for all interactions > 0.10).

**TABLE 2:** Associations between cognitive reserve and transitions across cognitive states and death by age and *APOE* genotypes.

Strata by age and	Hazards ratio (95% confidence interval), transitions			
*APOE* genotype	*n/N*	From normal cognition to dementia	*n/N*	From CIND to death
Age groups				
60‐72 years	30/1612	–	113/1612	0.65 (0.54‐0.77)^‡^
≥78 years	66/1019	–	205/1019	0.87 (0.75‐1.01)
*P* for interaction		0.353		0.010
*APOE* genotype^a^				
Any ε4 allele	60/1744	0.71 (0.48‐1.05)	188/1744	–
No ε4 allele	33/722	1.07 (0.85‐1.34)	93/722	–
*P* for interaction		0.051		0.332

*Note*: *n/N* indicates the number of transitions/the number of study participants.

Models were controlled for continuous age (years), sex, current smoking, heavy alcohol drinking, hypertension, high total cholesterol, diabetes, body mass index, atrial fibrillation, ischemic heart disease, and heart failure, and if applicable, for *APOE* genotypes.

Abbreviation: *APOE, apolipoprotein E*; CIND, cognitive impairment, no dementia.

^‡^ *P *< 0.001.

^a^ The number of participants with missing values was 165 for *APOE* genotypes.

## 4 DISCUSSION

The main findings from this population‐based long‐term cohort study can be summarized into the following points: (1) CR was linked to a reduced risk of transitions from normal cognition to CIND or death and from CIND to death. The reduced risk of progression from normal cognition to CIND or death could not be attributable to MRI markers of cerebral atrophy and microvascular damage, whereas the increased risk of transition from CIND to death was no longer evident; (2) CR was not evidently associated with the transition from CIND to dementia or from CIND to normal cognition; and (3) CR was associated with a lower risk of transition from CIND to death among individuals aged 60‐72 years, but the reduced risk of the transition was not evident among those aged 78 years and above.

The association of CR with lower risk of MCI has been previously reported in cohort studies.^5^ Our study provides evidence supporting the association of CR with lower risk of transition from normal cognition to CIND, a broader definition that included individuals who meet the criteria for MCI as well as those who are cognitively impaired but do not meet all the criteria for MCI.^25^ It has been proposed that enhanced neural connectivity and neural compensation are potential mechanisms underlying the cognitive advantages associated with CR.^26^, ^27^ A prospective cohort study from Germany found that CR, representing the cognitive variability not accounted for by demographic factors, genetic risks, and neuropathological burdens, was linked to increased intrinsic connectivity within brain networks, indicating a higher level of CR.^27^ In addition, a case‐control study of 16 healthy individuals, 12 patients with amnestic MCI, and 16 patients with mild Alzheimer's disease indicated that individuals with a higher level of CR, compared to those with a lower level of CR, displayed more actively engaged compensatory networks within healthy brain regions to sustain cognitive performance in the presence of brain pathology.^26^

We further showed that the association of CR with progression from normal cognition to CIND was present independent of MRI markers of cerebral atrophy and microvascular damage. This finding aligns well with the report from a systematic review.^5^ Prior studies only controlled for GM volumes as a marker of cerebral atrophy to address neuropathological impact,^14^ whereas we also controlled for MRI markers of microvascular damage, which is important given the potential role of microvascular damage in contributing to cognitive deterioration and the development of dementia.^15^, ^16^ Altogether, these findings suggest that, at a given load of brain pathology, high CR may help maintain cognitive function, particularly during the preclinical stage of dementia.

Notably, while the main effect of lifelong composite CR on cognitive transitions was most apparent during the predementia stages, the potential interaction of CR and *APOE*‐ε4 status was most visible for transitions from normal cognition to dementia. The ability of CR to mitigate dementia risk attributable to *APOE*‐ε4 allele has been reported previously,^19^ and our findings extend the previous work by simultaneously taking into account other transitions across the cognitive continuum, as well as death. Together, these results suggest that individuals with an inherited predisposition to dementia may also experience cognitive benefits from possessing a high level of CR.

We found an association of lifelong CR with a reduced risk of transition from normal cognition to CIND, but not with the transition from normal cognition to dementia neither with the transition from CIND to dementia. The limited statistical power might partly contribute to the lack of association of CR with the transition from normal cognition to dementia (*n* = 96). Similarly, data pooled from six cohort studies showed that higher educational attainment, as a CR indicator, was associated with a reduced risk of transition from normal cognition (MMSE score ≥27) to MCI (MMSE score range: 23‐26). However, educational attainment was not associated with transition from mild to severe cognitive impairment (MMSE score ≤22).^28^ Given that previous studies have linked CIND with substantial risk for dementia,^29^, ^30^ the association of CR with reduced risk of dementia is at least partly due to the delayed transition from normal cognition to CIND, rather than from CIND to dementia. The lack of association of CR with transition from CIND to dementia was consistent with the reports from the project of Biomarkers of Cognitive Decline Among Normal Individuals.^31^ Indeed, it has been hypothesized that CR may help maintain cognitive function until a threshold of brain pathology and that beyond which, CR may no longer provide the same level of cognitive benefit and individuals engaged in CR‐enhancing activities may even experience more rapid cognitive decline.^32^, ^33^

We also found that a high composite CR was related to a reduced risk of transition from normal cognition or CIND to death. This is in line with the findings from the Rotterdam study that CR is related to a lower mortality.^34^ One possible explanation is that individuals with higher CRs are more actively engaged in leisure activity and social networks, and physically healthier; both factors have been associated with a reduced risk of chronic diseases and depression,^35^, ^36^ which could contribute to lower mortality.^37^

In people with CIND initially, the association of CR with lower mortality rates was evident in young‐old (60‐72 years) but not in old‐old (≥78 years) individuals. This suggests that the beneficial effects of CR on survival may be more pronounced in the earlier stages of cognitive decline or impairment. Old‐old individuals, compared to young‐old people, usually have more advanced stages of cognitive impairment and brain pathology that are associated with increased mortality.^38^, ^39^

The major strength of our study is the population‐based design with a long‐term follow‐up period (up to 15 years; mean 9.7 years). Additional strengths include the integration of comprehensive data on cognitive phenotypes with brain MRI data to capture both cerebral atrophy and microvascular damage in a subsample, and the use of Markov multistate models to assess transitions across the cognitive continuum and death. However, there are also limitations to our study. First, due to the observational nature of our cohort study, caution is needed when inferring the causal relationship between CR and cognitive phenotypes. Second, some structural brain MRI markers of microvascular damage (e.g., cerebral microbleeds and microinfarcts) and functional MRI markers (e.g., blood oxygen level and functional connectivity) were not available due to the lack of relevant MRI sequences and limited spatial resolution, neither did we have access to direct measures or biomarkers of neurodegeneration. Third, other conceptual models of resilience, for instance, brain reserve or brain maintenance, could underpin some of the effects described here, and future studies ought to consider them jointly in larger study samples. Fourth, according to the SNAC‐K follow‐up schedule, we assessed cognitive states at different time intervals depending on baseline age of the study participants. This might have resulted in an underestimation of the associations between CR and transitions across cognitive states and death in the old‐old cohort. Last, the SNAC‐K participants were from central Stockholm who had a higher socioeconomic status than the national average, which should be kept in mind when generalizing our findings to other populations. In addition, the MRI subsample was relatively younger and healthier compared to the whole SNAC‐K sample, which may have resulted in an underestimation of the true associations between brain measures and health outcomes as well as the risk and benefit of transitions across cognitive states and death.

In conclusion, our long‐term population‐based prospective cohort study suggests that CR contributes to maintaining cognitive health in the prodromal phase of dementia, regardless of burdens of brain pathology that includes both atrophy and microvascular damage. In addition, in people with CIND, CR is associated with a lower risk of mortality in young‐old but not old‐old individuals. These findings may have important implications for the development of interventions to promote cognitive health in aging populations.

## CONFLICT OF INTEREST STATEMENT

The authors declare no conflicts of interest. Author disclosures are available in the supporting information.

## CONSENT STATEMENT

Written informed consent was obtained from all participants or, in case of cognitively impaired individuals, from next‐of‐kin.

## Supporting information

Supporting Information

Supporting Information
