# Supplementary material for: Association of cognitive reserve with transitions across cognitive states and death in older adults: A 15‐year follow‐up study
Source: Alzheimers Dement. 2024 May 23;20(7):4737–46. doi: 10.1002/alz.13910 (PMC11247709; doi:10.1002/alz.13910)
Supplement: Supplementary file 1 — Supporting Information [file ALZ-20-4737-s002.docx]

**Supplementary materials**

**Association of cognitive reserve with transitions across cognitive states and death in older adults: A 15-year follow-up study**

Li Y, et al.

**Contents**

**Supplemental Table 1.** Characteristics of study participants by cognitive impairment, no dementia at baseline in the SNAC-K MRI subsample (n=517)

**Supplemental Figure 1.** Standardized estimates for the composite cognitive reserve score derived from the structural equation models in the SNAC-K total sample (n=3060)

**Supplemental Figure 2.** The correlations of composite cognitive reserve score with (A) gray matter volume, (B) hippocampal volume, (C) white matter hyperintensity volume, and (D) count of perivascular spaces (n=517).

**Supplemental Figure 3.** Total numbers of transitions across cognitive states and death in (A) the SNAC-K total sample and (B) the SNAC-K MRI subsample

**Supplemental Figure 4.** Association of individual cognitive reserve indicators with transitions across cognitive states and death in the SNAC-K total sample (n=2631)

**Supplemental Table 1.** Characteristics of study participants by cognitive impairment, no dementia at baseline in the SNAC-K MRI subsample (n=517)

| **Characteristics** | **Total sample,** | **Cognitive impairment, no dementia** | | |
| --- | --- | --- | --- | --- |
|  | n=517 | No, n=417 | Yes, n=100 | *P-*value |
| **Age**, years | 71.13 (9.08) | 70.87 (9.17) | 72.24 (8.64) | 0.176 |
| **Female**, n (%) | 304 (58.80) | 239 (57.31) | 65 (65.00) | 0.161 |
| **Education^a^**, years | 12.65 (4.07) | 12.95 (4.03) | 11.41 (4.02) | <0.001 |
| **Midlife work complexity score^a^** | 5.13 (1.80) | 5.21 (1.84) | 4.79 (1.56) | 0.035 |
| **Late-life leisure activity score^a^** | 2.69 (1.46) | 2.75 (1.47) | 2.43 (1.40) | 0.059 |
| **Late-life social network score^a^** | 0.83 (0.49) | 0.15 (0.48) | 0.05 (0.55) | 0.054 |
| **Composite cognitive reserve score** | 0.21 (1.25) | 0.29 (1.26) | -0.15 (1.16) | 0.001 |
| **Current smoking**, n (%) | 64 (12.38) | 51 (12.23) | 13 (13.00) | 0.834 |
| **Heavy alcohol** **drinking**, n (%) | 86 (16.63) | 73 (17.51) | 13 (13.00) | 0.277 |
| **Hypertension**, n (%) | 373 (72.15) | 296 (70.98) | 77 (77.00) | 0.228 |
| **Diabetes**, n (%) | 40 (7.74) | 25 (6.00) | 15 (15.00) | 0.002 |
| **High total cholesterol^a^**, n (%) |  |  |  | 0.023 |
| No | 223 (43.13) | 192 (46.04) | 31 (31.00) |  |
| Yes | 287 (55.51) | 220 (52.76) | 67 (67.00) |  |
| **Body mass index**, kg/m² | 26.03 (4.08) | 25.89 (4.06) | 26.58 (4.16) | 0.135 |
| ***APOE*** **genotypes^a^**, n (%) |  |  |  | 0.418 |
| No ε4 allele | 359 (69.44) | 293 (70.26) | 66 (66.00) |  |
| Any ε4 allele | 138 (26.69) | 110 (26.38) | 28 (28.00) |  |
| **Atrial fibrillation**, n (%) | 32 (6.19) | 26 (6.24) | 6 (6.00) | 0.930 |
| **Heart failure**, n (%) | 32 (6.19) | 21 (5.04) | 11 (11.00) | 0.026 |
| **Ischemic heart disease**, n (%) | 63 (12.19) | 46 (11.03) | 17 (17.00) | 0.101 |

Abbreviations: SNAC-K, The Swedish National study on Aging and Care in Kungsholmen; MRI, magnetic resonance imaging; *APOE*, apolipoprotein E gene.

Data were mean (SD), unless otherwise specified.

^a^ The number of participants with missing value was 3 for education, 4 for midlife work complexity score, 28 for late-life leisure activity score, 13 for later-life social network score, 7 for high cholesterol, and 20 for *APOE* genotypes.


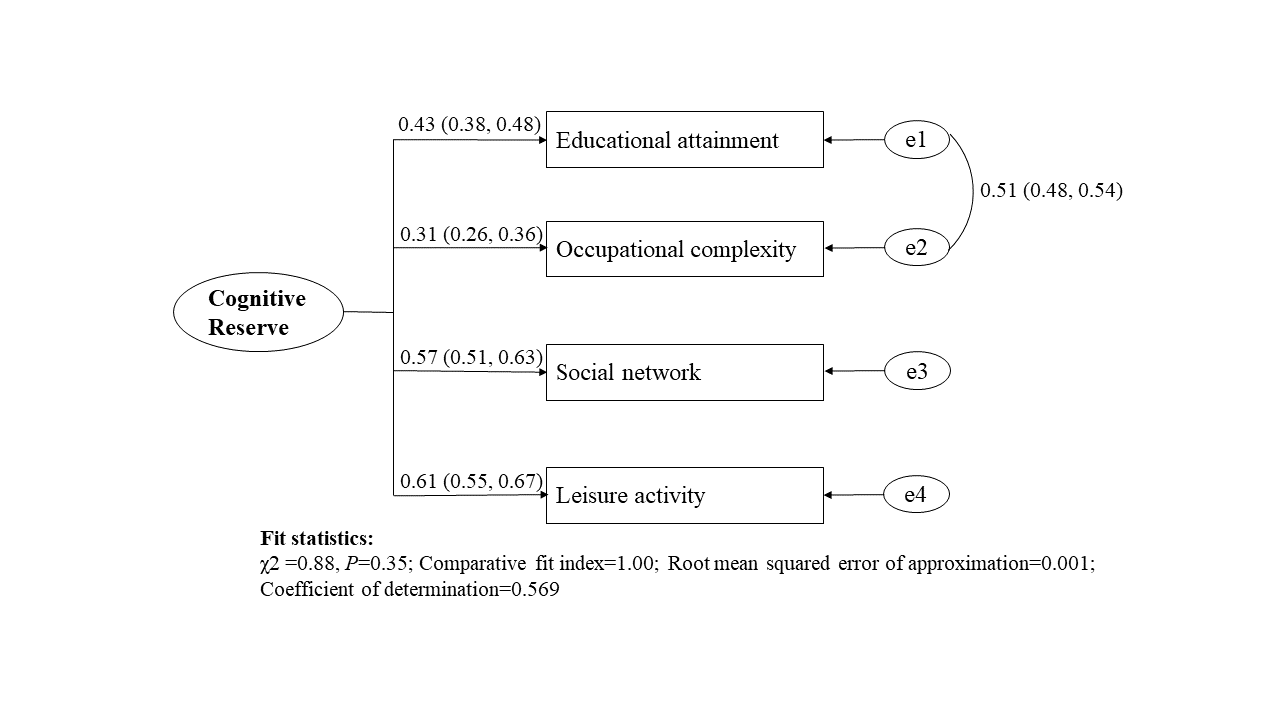


**Supplemental Figure 1.** Standardized estimates for the composite cognitive reserve score derived from the structural equation models in the SNAC-K total sample (n=3060).

The values indicate the β-coefficients (95% confidence intervals) of the four observable factors over the life course used to generate the composite score of cognitive reserve from the structural equation models.

e1, e2, e3, and e4 represent the measurement error for each of the four observable factors in estimating the composite cognitive reserve score.

| A. Gray matter volume  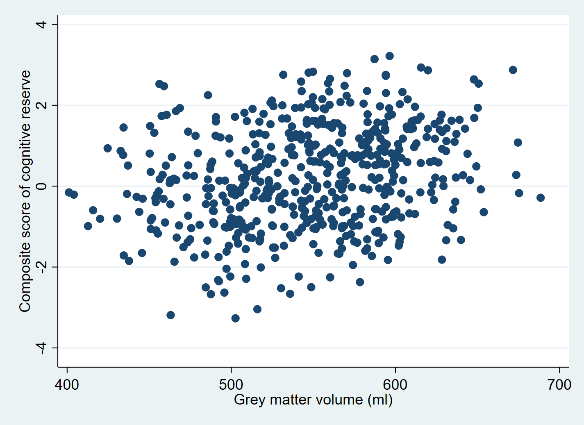 | B. Hippocampal volume  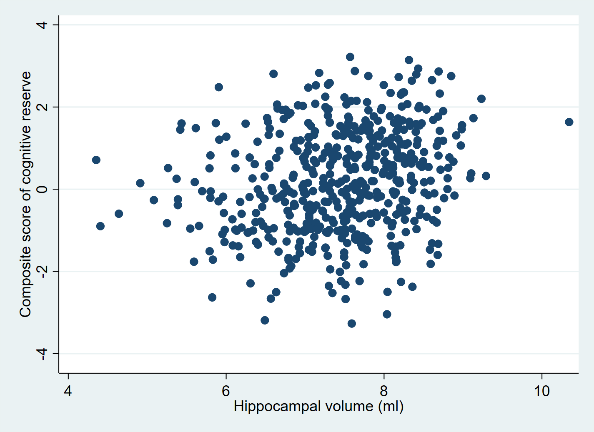 |
| --- | --- |
| C. White matter hyperintensity volume  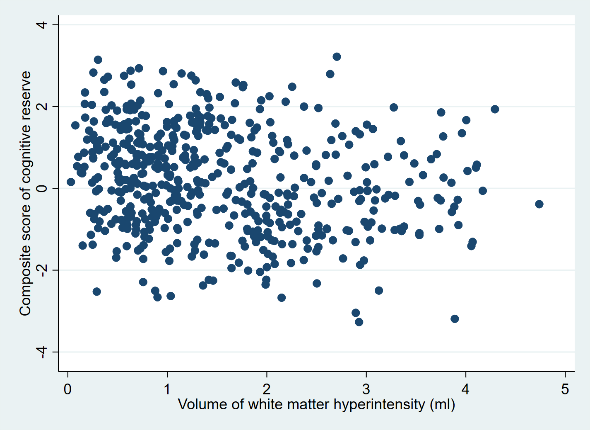 | D. Count of perivascular spaces  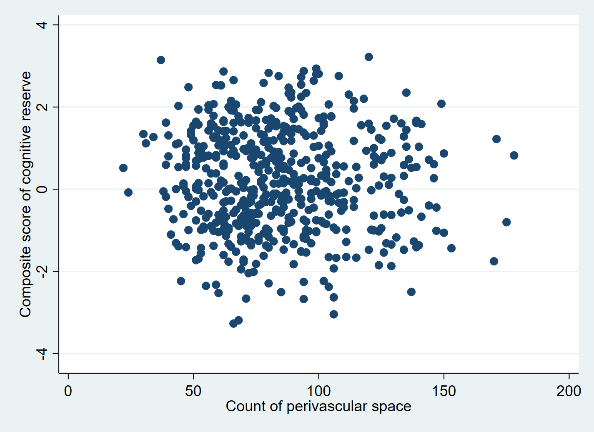 |

**Supplemental Figure 2.** The correlations of composite cognitive reserve score with (A) gray matter volume, (B) hippocampal volume, (C) white matter hyperintensity volume, and (D) count of perivascular spaces (n=517).

A. Transitions across cognitive states and death in the SNAC-K total sample (n=2631)

**Cognitive impairment, no dementia**

**Normal cognition**

**Dementia**

n=528

n=89

n=318

n=591

.80, 0.94)^‡^

n=124

n=96

**Death**

n=153

B. Transitions across cognitive states and death in the SNAC-K MRI subsample (n=517)

**Cognitive impairment, no dementia**

**Normal cognition**

**Dementia**

n=108

n=15

n=45

n=126

n=20

**Death**

n=42

n=25

**Supplemental Figure 3.** The numbers of participants in transitions across cognitive states and death in (A) the SNAC-K total sample and (B) the SNAC-K MRI subsample

Abbreviations: SNAC-K, The Swedish National study on Aging and Care in Kungsholmen; MRI, magnetic resonance imaging.

**Cognitive impairment, no dementia**

**Normal cognition**

**Dementia**

**Death**

edu: 1.02 (0.96, 1.08)

occ: 1.16 (1.01, 1.32)^*^

soc: 1.09 (0.71, 1.69)

lei: 1.02 (0.90, 1.17)

edu: 0.96 (0.89, 1.04)

occ: 1.20 (0.99, 1.44)

soc: 1.18 (0.68, 2.04)

lei: 0.90 (0.75, 1.09)

edu: 0.91 (0.83, 0.99)^*^

occ: 1.26 (1.04, 1.53)^*^

soc: 0.77 (0.44, 1.36)

lei: 0.95 (0.77, 1.18)

edu: 1.03 (0.99, 1.06)

occ: 0.92 (0.87, 0.98)^*^

soc: 0.67 (0.55, 0.82)^†^

lei: 0.91 (0.85, 0.98)^*^

edu: 0.98 (0.95, 1.01)

occ: 0.86 (0.81, 0.92)^‡^

soc: 0.79 (0.64, 0.97)^*^

lei: 0.94 (0.88, 1.00)

edu: 1.01 (0.97, 1.06)

occ: 1.01 (0.92, 1.12)

soc: 0.50 (0.38, 0.67)^‡^

lei: 0.85 (0.76, 0.96)^†^

edu: 0.99 (0.93, 1.06)

occ: 0.90 (0.77, 1.05)

soc: 1.16 (0.68, 1.97)

lei: 0.86 (0.72, 1.02)

**Supplemental Figure 4.** Association of individual cognitive reserve indicators with transitions across cognitive states and death in the SNAC-K total sample (n=2631)

Data are hazards ratios (95% confidence intervals), derived from the Markov multi-state models, which represented risk of the transition associated with per 1-point increase in the score of each of the four cognitive reserve indicators (range: -4.25 – 3.46).

Abbreviations: edu: educational attainment; occ: occupational complexity; soc: social network; lei: leisure activity.

^*^*P*<0.05, ^†^*P*<0.01, ^‡^*P*<0.001.
